# Phenotypic and genotypic characterization of familial adult myoclonus epilepsy in a Chinese case series

**DOI:** 10.1093/braincomms/fcaf214

**Published:** 2025-06-04

**Authors:** Sheng Zeng, Yao Zhou, Yuwen Zhao, Mingqiang Li, Chaojun Zhou, Xuejing Wang, Hui Quan, Tiandong Che, Jinchen Li, Qiying Sun, Beisha Tang

**Affiliations:** Department of Geriatrics, The Second Xiangya Hospital, Central South University, Changsha, Hunan 410011, China; Department of Neurology, Xiangya Hospital, Central South University, Changsha, Hunan 410008, China; Department of Neurology, Xiangya Hospital, Central South University, Changsha, Hunan 410008, China; Department of Neurology, The First Affiliated Hospital of University of South China, Hengyang, Hunan 421001, China; Department of Neurology, The First Affiliated Hospital & Clinical Research Center for Immune-Related Encephalopathy of Hunan Province, Hengyang Medical School, University of South China, Hengyang, Hunan 421001, China; Department of Neurology, Changde Hospital, Xiangya School of Medicine, Central South University (The First People’s Hospital of Changde City), Changde City, Hunan 415000, China; Department of Neurology, The First Affiliated Hospital of Zhengzhou University, Zhengzhou 450052, China; Institute of Parkinson and Movement Disorder, Zhengzhou University, Zhengzhou, Henan 450052, China; Bioinformatics Center, Annoroad Gene Technology, Beijing 100176, China; Bioinformatics Center, Annoroad Gene Technology, Beijing 100176, China; Center for Medical Genetics, School of Life Sciences, Central South University, Changsha, Hunan 410008, China; Bioinformatics Center, National Clinical Research Center for Geriatric Disorders, Xiangya Hospital, Central South University, Changsha, Hunan 410008, China; Key Laboratory of Hunan Province in Neurodegenerative Disorders, Central South University, Changsha, Hunan 410008, China; Bioinformatics Center, National Clinical Research Center for Geriatric Disorders, Xiangya Hospital, Central South University, Changsha, Hunan 410008, China; Key Laboratory of Hunan Province in Neurodegenerative Disorders, Central South University, Changsha, Hunan 410008, China; Department of Geriatric Neurology, Xiangya Hospital, Central South University, Changsha, Hunan 410008, China; Department of Neurology, Xiangya Hospital, Central South University, Changsha, Hunan 410008, China; Department of Neurology, The First Affiliated Hospital & Clinical Research Center for Immune-Related Encephalopathy of Hunan Province, Hengyang Medical School, University of South China, Hengyang, Hunan 421001, China; Bioinformatics Center, National Clinical Research Center for Geriatric Disorders, Xiangya Hospital, Central South University, Changsha, Hunan 410008, China; Key Laboratory of Hunan Province in Neurodegenerative Disorders, Central South University, Changsha, Hunan 410008, China

**Keywords:** SAMD12, TTTCA repeat expansion, targeted long-read sequencing, phenotype-genotypic correlation

## Abstract

Familial adult myoclonus epilepsy is a type of repeat expansion disorders caused by insertion of the causative pentanucleotide TTTCA repeat into an intronic polymorphic TTTTA repeat in different genes. We aimed to characterize the clinical features and elucidate the exact genetic basis of TTTTA/TTTCA repeat expansion in familial adult myoclonus epilepsy from mainland China. Eighty-five individuals including 36 patients and 49 normal phenotype relatives from seven pedigrees with familial adult myoclonus epilepsy, were recruited in a case series from mainland China. Repeat-primed PCR was used for initial screening. Long-range PCR-based enrichment, followed by targeted deep HiFi long-read sequencing, was performed to precisely clarify the detailed information of causative pentanucleotide TTTTA/TTTCA repeat expansion. The results indicated there exists obvious clinical heterogeneity both within and between families in our patient group. All patients were genetically diagnosed with familial adult myoclonus epilepsy type 1. The number of pentanucleotide repeats was extremely unstable, with median TTTCA repeat sizes ranging from 10 to 647 in the affected members of our case series under a mean sequence depth of coverage above 50 000. The [(TTTTA)exp (TTTCA)exp] motif was the only configuration of expanded *SAMD12* repeats in our case series. An inverse correlation was found between the age of onset and the number of TTTCA repeats and the total number of TTTTA/TTTCA repeats. Clinical anticipation was observed for tremor and seizure symptoms. However, we did not demonstrate a link between parent-offspring differences in repeat sizes and their changes in age of onset. In summary, we determined the nature of the expanded repeats and a reliable phenotype-genotypic correlation in our case series of familial adult myoclonus epilepsy through targeted deep HiFi long-read sequencing technologies.

## Teaser

Zeng et al. characterize 36 patients with familial adult myoclonus epilepsy type 1 using HiFi long-read sequencing. They delineated clinical features, identified highly unstable expanded *SAMD12* TTTTA/TTTCA repeats, and demonstrated an inverse correlation between repeat size and age of onset. The study advances the understanding of this disorder.

## Graphical Abstract

**Graphical Abstract:**
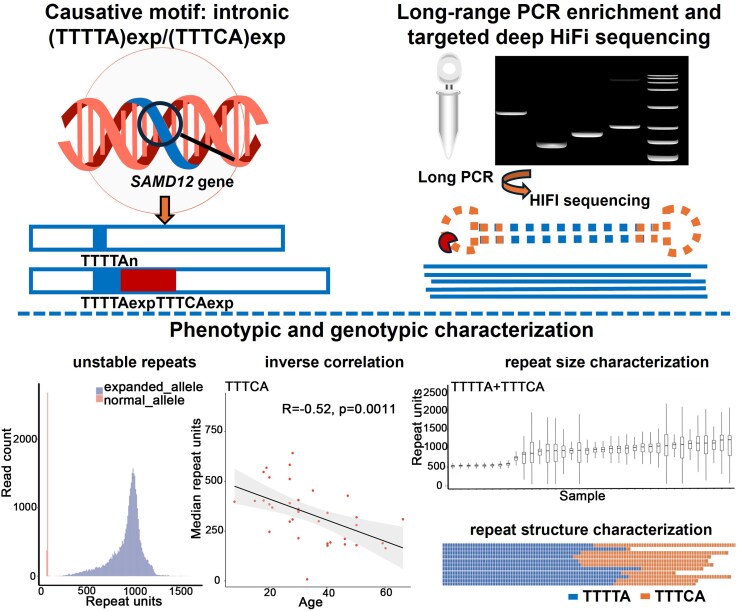

## Introduction

Familial adult myoclonus epilepsy (FAME) is an adult-onset autosomal dominant disorder characterized by cortical tremor and infrequent generalized epileptic seizures due to an inserted causative pentanucleotide TTTCA repeat into an intronic polymorphic TTTTA repeat in different genes.^1,2^ FAME was recognized under various other names including ‘autosomal dominant cortical myoclonus and epilepsy’, ‘familial cortical myoclonic tremor with epilepsy’, ‘benign adult familial myoclonic epilepsy’, etc. since 1985.^3^ Seven subtypes involving seven genes (*SAMD12*, *STARD7*, *MARCH6*, *YEAST2*, *TNRC6A*, *RAPGEF2* and *RAI1*) have been reported,^1,4-7^ with FAME1 (*SAMD12*) being the most common. As one of the repeat expansion disorders, there is strong clinical and genetic heterogeneity in FAME. Phenotypic and genotypic characterization is important for a deeper understanding of FAME. Additionally, it is necessary to elucidate the correlation between causally expanded *SAMD12* repeats and clinical manifestations. Several reports have pierced this issue and obtained different conclusions.^1,8-10^ An important reason for this phenomenon is the challenge of clarifying the precise sequence and structural information of the large expanded repeat element.

Elucidation of the exact genetic basis of TTTTA/TTTCA repeat expansion is necessary. However, the complete sequence span of this target region remains challenging because the huge expansion is beyond the scope of conventional genetic methods, such as Sanger sequencing and next-generation sequencing-based technologies. In addition, the depth of sequence is of equal importance, as potential somatic mosaicism can contribute to bias of repeat number. Here, we performed deep-targeted PacBio HiFi long-read sequencing (LRS) following long-range PCR (LR-PCR)-based enrichment to determine the nature of the expanded TTTTA/TTTCA repeats in our case series.

## Materials and methods

### Subjects

**Figure 1:**
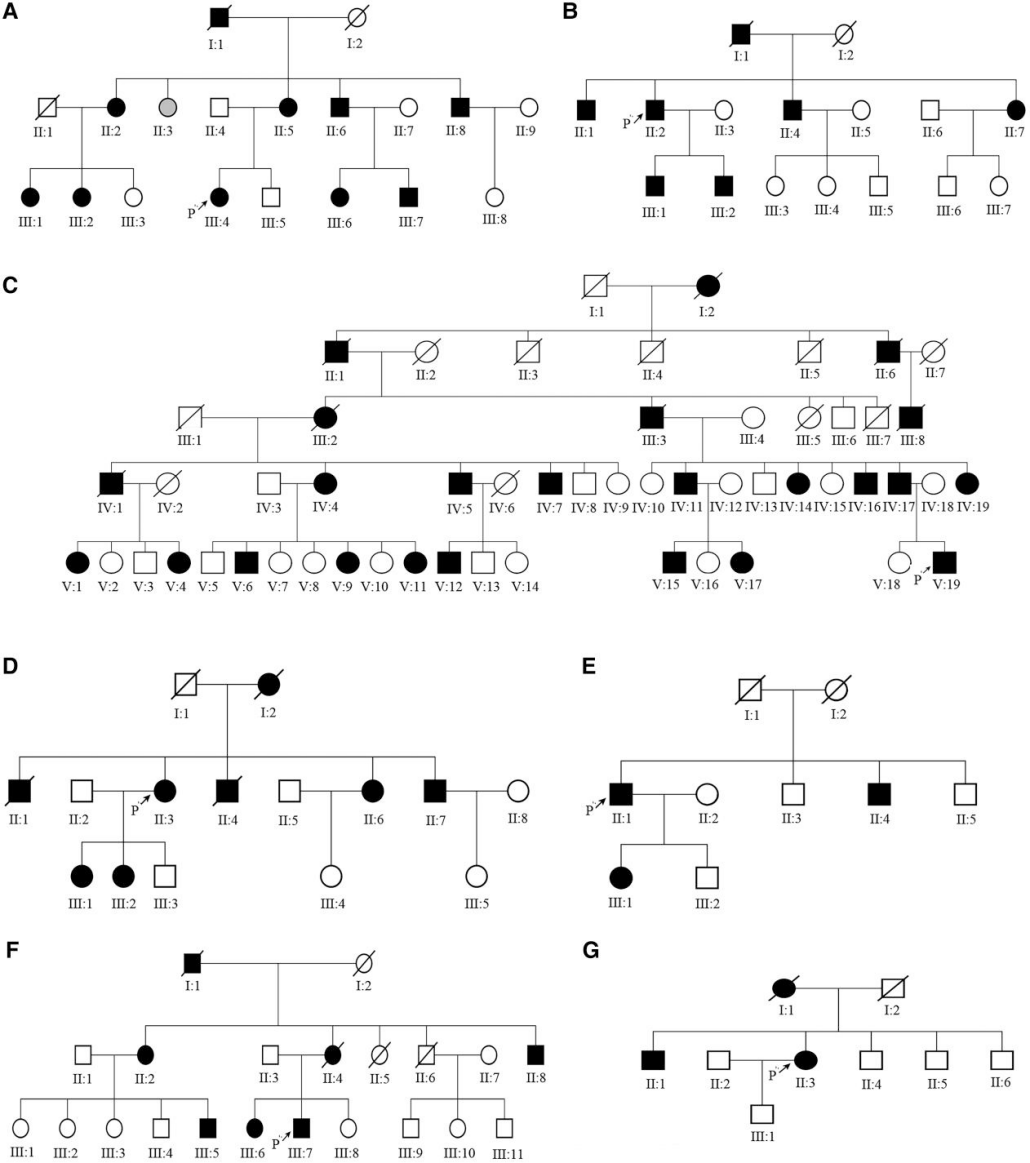
**Pedigrees.** (**A–G**): Seven pedigrees were analysed in our study, P, proband.

Eighty-five individuals (36 cases and 49 normal phenotype relatives) from seven pedigrees (Fig. 1A–G) were recruited for our study at Xiangya Hospital, Central South University. All affected individuals were subjected to a medical history review and thorough neurological examination by two or more experienced neurologists. Laboratory tests, brain imaging, and electrophysiological examinations were performed, and clinical data were collected. The diagnosis was made according to the criteria proposed in 2005.^3^ The study was approved by the Ethics Committee of Xiangya Hospital of Central South University in China (equivalent to an Institutional Review Board), and written informed consent was obtained from all participants. After obtaining informed consent, blood samples were obtained and genomic DNA was extracted from peripheral blood using standard extraction methods. Among the seven pedigrees, family C was reported in our previous study.^11^

### Methods

#### Repeat-primed PCR

All individuals were screened using repeat-primed PCR (RP-PCR). Briefly, for each DNA sample, fluorescent PCR was performed using a set of three primers: a fluorescently labelled locus-specific primer, a TTTTA or TTTCA repeat-specific primer with a random DNA sequence at the 5′ end, and a universal primer containing the same 5′ sequence as the repeat primer (sequence of primers according to reference^1^). The fluorescent PCR products were separated by capillary electrophoresis on an ABI Prism 3730 Genetic Analyzer and the data were examined using GeneMapper software (Applied Biosystems, Vernon Hills, IL, USA). Expansion of the pentanucleotide repeat revealed a sawtooth pattern.

#### LR-PCR-based enrichment

Individuals that tested positive by RP-PCR were further subjected to LR-PCR. LR-PCR was performed using primers and procedure according to reference.^12^ Specific barcodes for each sample were also added to the primers to guarantee the uniqueness of each LR-PCR reaction. The PCR products were separated by electrophoresis on a 1% agarose gel with a DNA marker. Expansion of the pentanucleotides repeat revealed an abnormally large PCR product. Allele dropout during amplification was observed between the PCR products of the normal and expanded sequences. To minimize sequence bias, we selectively decreased the product abundance of the normal allele by using a DNA selection bead system. All PCR products of the positive samples revealed by LR-PCR were mixed in the next sequencing step.

#### Targeted HiFi LRS

AMPure PB beads were used to purify of the pools of mixed PCR products. A SMRT bell library was constructed using the Pacific Biosciences SMRT bell express template prep kit 3.0. The constructed library was selected for fragment size on the BluePippin™ system, constructed with an insertion fragment size of 15 kb, and then subjected to primer annealing and bound the SMRT bell template to the polymerase using a DNA polymerase binding kit. The library was run on a PacBio Sequel SMRT cell. Raw subreads were obtained and processed using a circular consensus sequencing workflow (PacBio SMRT Link version 10.1) to generate clean HiFi reads. The HiFi reads were then aligned to the primary assembly of human reference genome GRCh38 using the minimap2.^13^ The aligned data were further demultiplexed to separate reads according to the specific barcode. To identify the exact size of each sample, we first screened and kept the reads covering the repeat region completely by detection of double-ended flanking sequence. Regular expression was used to calculate the number of TTTTA and TTTCA motifs for each selected read sequence. Expanded alleles were distinguished by the detection of TTTCA insertion. The mean, median and mode values of the TTTTA, TTTCA and TTTTA + TTTCA motifs were calculated for each sample. The size, configuration and composition of the *SAMD12* repeats of the expanded alleles were analysed.

#### Long-read whole-genome sequencing

DNA samples of four members (II2, II5, II6 and II8) from family a were also sequenced using the PromethION nanopore sequencer of the Oxford Nanopore Technologies platform. Library preparation was performed using a 1D Genomic DNA ligation kit (SQK-LSK109) according to the manufacturer's protocol. The DNA library was then loaded onto a PRO-002 (R9.4.1) flow cell and sequenced. The raw data base-calling was performed using guppy v.3.3.0 (Oxford Nanopore Technologies), and only pass reads (Qscore ≥ 7) were used for subsequent analysis. For each sample, the repeat number of each read that aligned with the short tandem repeat (STR) locus of human reference genome was detected using RepeatHMM (https://github.com/WGLab/RepeatHMM.git), as described previously.^11^

#### Statistical analysis

All statistical analysis were performed using Python (version 3.8) software. Pearson's correlation between the age at onset and expanded *SAMD12* repeats was calculated. The relationship between repeat size and its volatility, age at visit and volatility of repeat size was also evaluated. We also compared the occurrence ratio of clinical anticipation, differences in intergenerational dynamic expansion of the repeats, repeat sizes and changes in age of onset (AO) between maternal and paternal transmissions. Statistical *P* values was set at *P* < 0.05.

## Results

### Clinical characteristics of FAME in our case series

A total of 36 patients (19 males and 17 females) from seven families were included in the clinical analysis. The mean age of the patients was 53 years (range, 19–77 years). In our case series, features varied among patients, both within and between families. Generally, 14 and 3 patients manifested only cortical myoclonic tremor (CMT) and generalized tonic and clonic seizures (GTCS), respectively, whereas 19 patients had both CMT and GTCS. CMT was the first symptom in 91.7% of the affected individuals (33/36), with a mean AO of 34 years (range, 15–60 years). Seizures preceding CMT were observed in three patients (8.3%), and seizures were present in 22 patients with a mean AO of 36 years (range, 8–60 years). The severity of CMT and GTCS varied among the patients. Other accompanying symptoms, including mental and cognitive dysfunction, were observed in several cases (4/36). In addition, clinical anticipation was observed in parent-child pairs of our families. Some patients underwent brain imaging and electrophysiological testing. Brain MRI revealed no abnormalities in all ten patients examined, and interictal EEG demonstrated epileptiform discharges in all seven patients tested. Recorded somatosensory evoked potentials indicated enhanced cortical components by stimulating the median nerve bilaterally in the wrist [amplitude >10 µV (N20-P25) bilaterally, Supplementary Fig. 1]. Tremor analysis of the electromyogram in a patient revealed irregular tremor with a frequency of 8.5 Hz. The clinical features of the patients are summarized in Table 1.

**Table 1:** Clinical data of patients

Pedigree: ID	Gender	Age	CMT	Seizure		Psychomotor development	Cognitive dysfunction
			Onset age	Onset age	Frequency^a^		
A: II2	F	76	60s		/	N	
A: II5	F	70	59	60s	+	N	
A: II6	M	67	40s		/	N	
A: II8	M	56	46		/	N	
A: III1	F	52	50		/	N	
A: III2	F	50	40		/	N	
A: III4	F	44	30	42	++	N	
A: III6	F	43	41		/	N	
B: II1	M	66	35	30	+	N	
B: II2	M	63	40	20	++	N	
B: II4	M	60	30	40	+	N	
B: II7	F	57		8s	+	N	
B: III1	M	39	20	35	+	N	
B: III2	M	36	18	37	+	N	
C: IV4	F	77	50s	50s	+	N	
C: IV5	M	68	27		/	N	
C: IV7	M	62	30		/	N	
C: IV11	M	61	27	27	++	N	
C: IV14	F	54	37	37	+	N	
C: IV17	M	45	35	35	+	N	
C: IV19	F	40	40	40	+	N	
C: V9	F	43	30		/	N	
C: V11	M	37	21	37	+	N	
C: V12	M	43	28	40	+	Learning disabilities	
C: V15	M	37	18		/	N	
C: V19	M	19	15		/	N	
D: II3	F	74	66	66	+	N	Mild impairment
D: II7	M	54	50	50	+	N	
D: III1	F	50	47	47	+	N	
E: II1	M	66	46		/	N	
E: III1	F	34	20		/	N	
F: II2	F	75		27	++	N	Moderate impairment
F: III5	M	42		27	+	N	
F: III6	F	49	19	19	+	N	Mild impairment
F: III7	M	43	33	28	+	N	
G: II3	F	38	33		/	N	

CMT, cortical myoclonic tremor; N, normal; /, data unavailable.

^a^Grade of seizure frequency: +, rare (≤1/year); ++, occasional (1–6/year); +++, frequent (>6/year).

### Genetic analysis of FAME in case series

**Figure 2:**
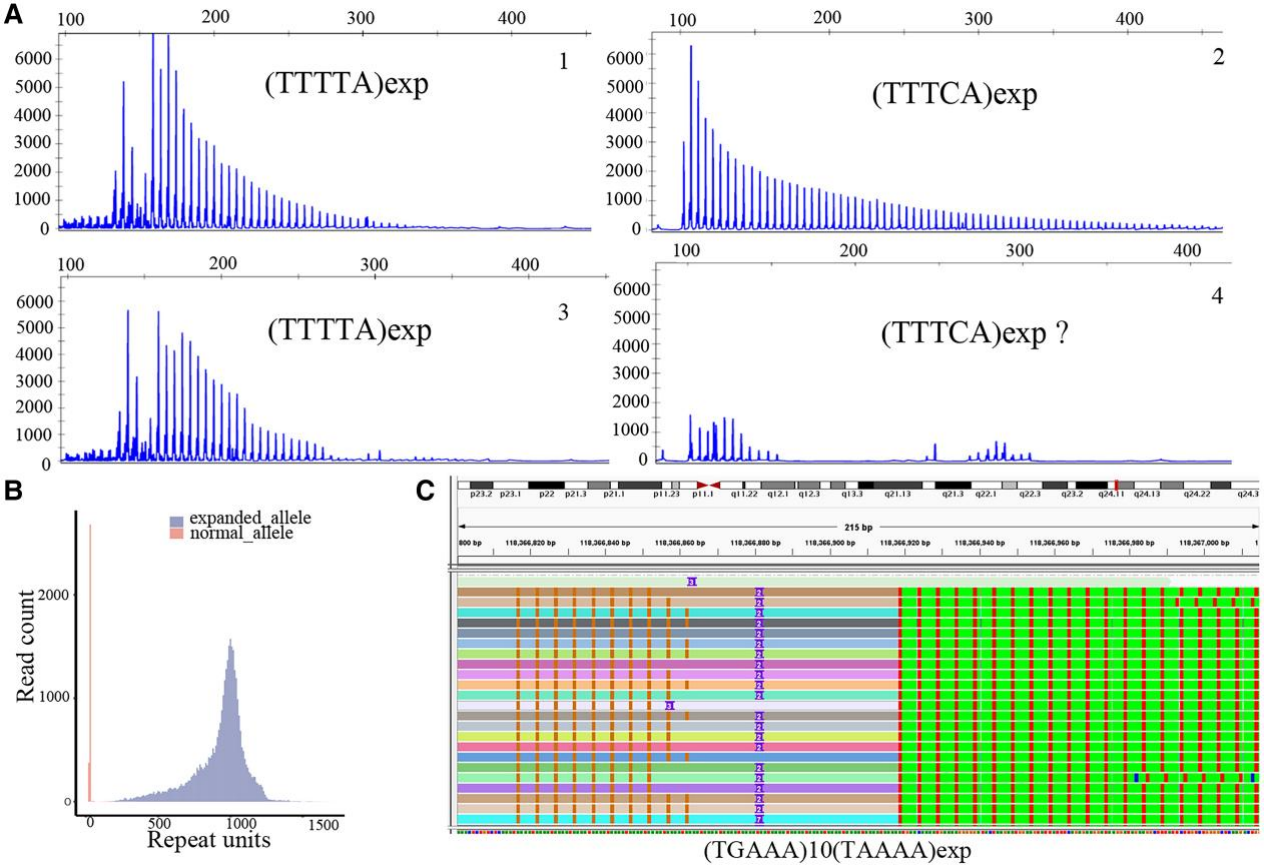
**Representative results of repeat detection**. (**A**) Representative results of RP-PCR. 1–2 indicated typical positive TTTTA and TTTCA expansion signals, respectively. 3–4 presented typical positive TTTTA and indistinguishable TTTCA expansion signal, respectively. (**B**) A representative histogram showing remarkably unstable repeats (TTTTA + TTTCA) with an approximately normal distribution. (**C**) Patient G: II3 had an extremely short TTTCA insertion (10 TTTCA repeat units), which would explain the indistinguishable signal presented in RP-PCR. exp, expansion.

**Figure 3:**
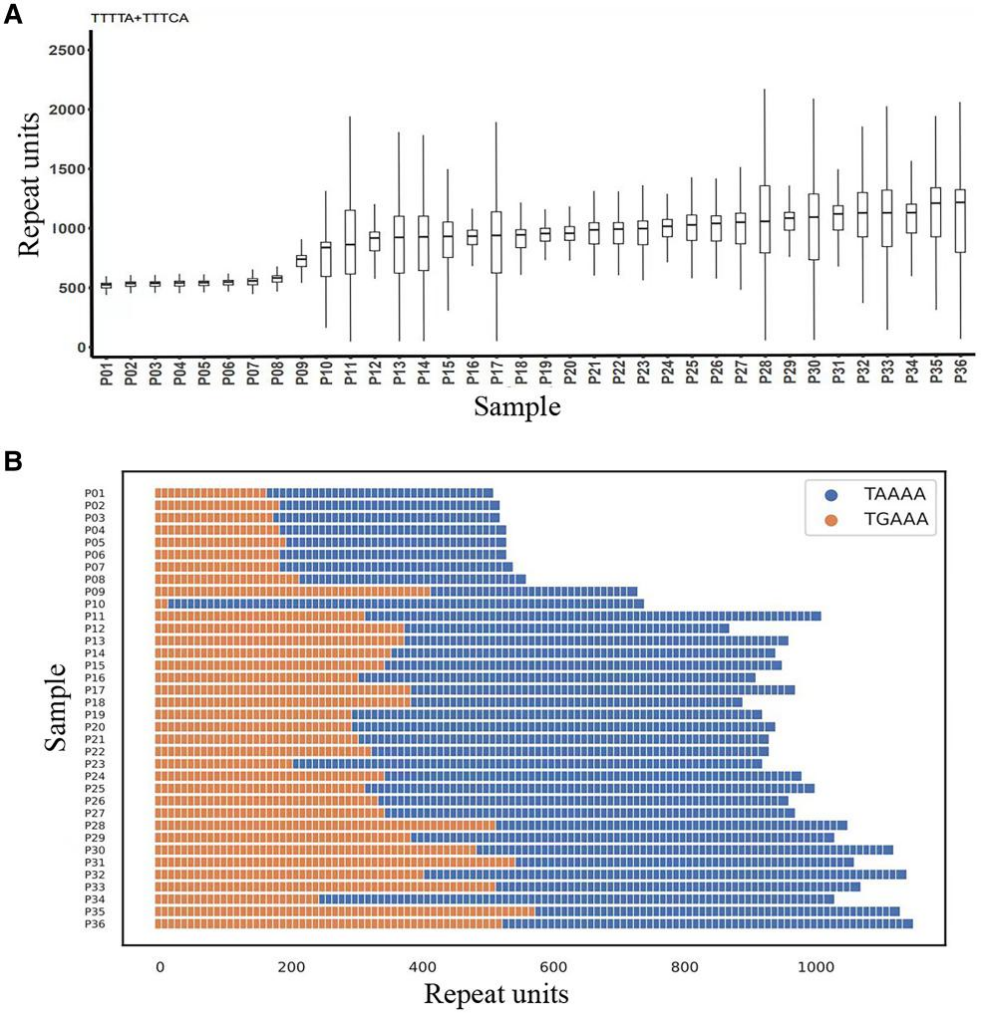
**Sequence and structure information of the expanded repeat element.** (**A**) The box plot indicates that the expanded repeats were unstable, with median TTTTA + TTTCA repeat numbers ranging from 526 to 1226; Box = interquartile range (IQR), bold horizontal line = median, whiskers = upper quartile + 1.5×IQR and lower quartile-1.5×IQR. (**B**) [(TTTTA)exp(TTTCA)exp] configuration was the only type of repeat composition, and the proportion of the pathogenic TTTCA repeats in the overall repeat sequence ranged from 1.2% to 62.6% in our case series. TGAAA and TAAAA were the target sequences of the opposite strand. exp, expansion.

**Figure 4:**
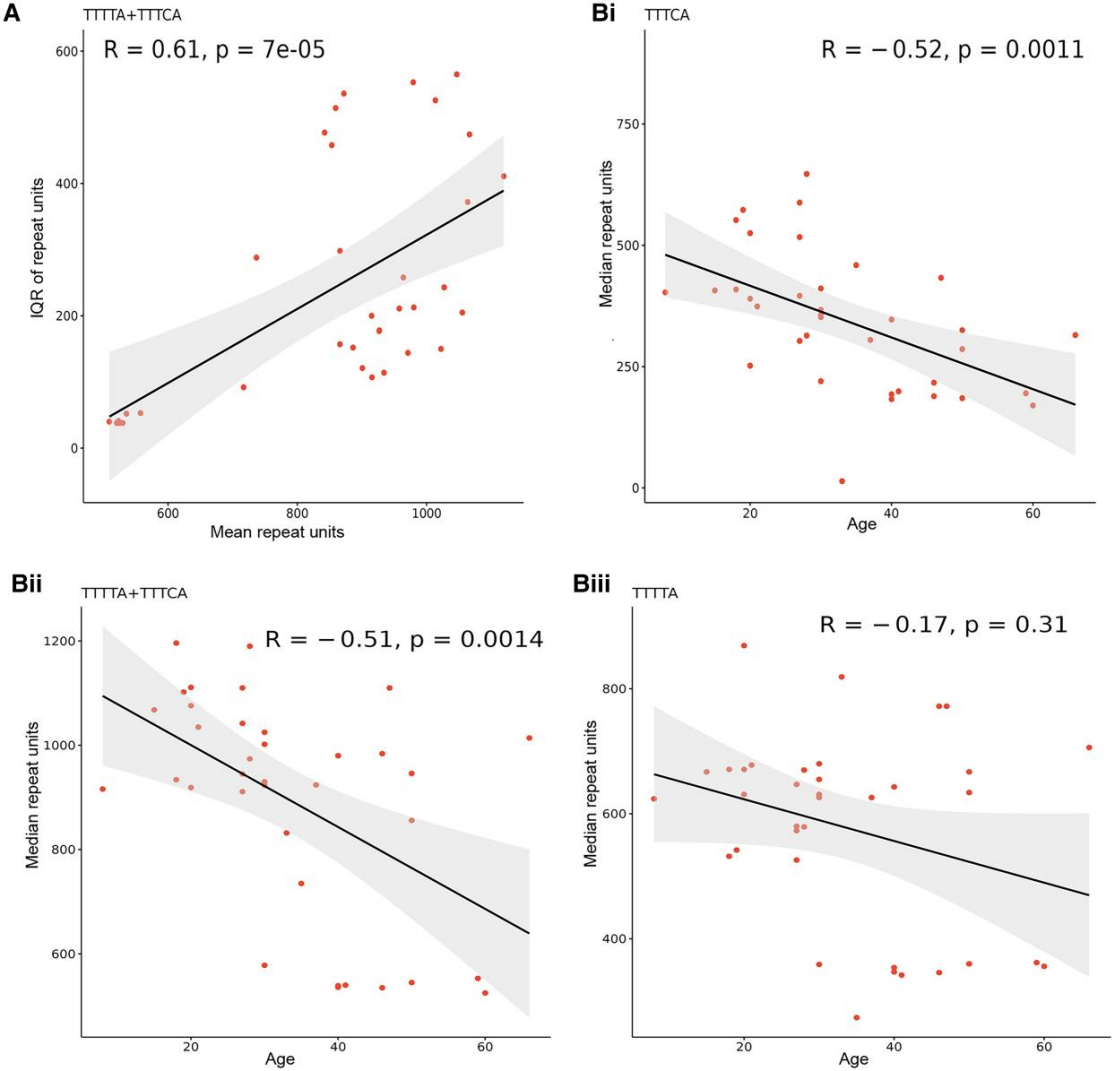
**Structural information of the expanded repeat element**. Scatter plot and linear regression of correlation analysis. (**A**) Repeat instability was related to its average number (Pearson's, *N* = 36, *R* = 0.61, *P* = 7e−05). Each data point represents the repeat number difference of a sample, calculated as the interquartile range (IQR) of the total expanded TTTTA/TTTCA repeats. (**B**) A significant inverse correlation between the median length of TTTCA and AO (Pearson's, *N* = 36, *R* = −0.52, *P* = 0.0011, **Bi**) and between the median length of TTTTA/TTTCA and AO (Pearson's, *N* = 36, R = −0.51, *P* = 0.0014 **Bii**). No correlation was found between the length of TTTTA and AO (Pearson's, *N* = 36, *R* = −0.3, *P* = 0.078 **Biii**).

Eighty-five individuals were subjected to an initial screen of TTTTA and ‘inserted’ TTTCA expansion in *SAMD12*. As a result, 35 case individuals showed definite positive signals (Fig. 2A-1 and 2), and one case (G: II3) presented an indistinguishable TTTCA expansion signal (Fig. 2A-3 and 4). Abnormal large PCR products with sizes ranging from 3 to 10 kb were identified in all 36 case individuals by LR-PCR (Supplementary Fig. 2). To gain better insights into repeat configurations and compositions, we sequenced 36 individuals using combined LR-PCR-based enrichment with high-depth PacBio HiFi LRS. A total of 80 Gb of data were produced, with an average data of 2.2 Gb (0.4–6.8 Gb), an average total sequencing depth of 58 994 (7412–227 470) and an expanded allele sequencing depth of 29 246 (5756–82 120) for each individual from LRS. The repeat number was counted for polymorphic TTTTA and pathogenic TTTCA in the normal and expanded alleles of every read for all samples. We observed that the expanded repeats of TTTTA, TTTCA and TTTTA + TTTCA were remarkably unstable and presented an approximately normal distribution (Fig. 2B). We then separated the reads of expanded allele and defined three repeat modules, TTTTA, TTTCA and TTTTA + TTTCA, for further analysis. The mean, median and mode values were calculated for the three modules for all samples (Table 2). We initially evaluated the performance of the method of combined LR-PCR-based enrichment with HiFi LRS, using the four case individuals that were also subjected to whole-genome LRS. The causative SAMD12 repeats in the four cases were covered by an average sequencing depth of 15 (11–18) in the whole-genome LRS data. The repeat count of each read was determined by repeatHMM and further checked manually. Consistent with the findings from LR-PCR-based HiFi sequencing, unstable repeat numbers were also observed in whole-genome LRS data (Supplementary Table 1). The mean, median, and mode values were also calculated for the expanded repeats of TTTTA, TTTCA and TTTTA + TTTCA (Supplementary Table 2). The difference in the expanded TTTTA and TTTCA repeat numbers calculated between these two methods was small (0.4–1.1%) for the four individuals. Therefore, the strategy of combining LR-PCR-based enrichment with HiFi LRS was reliable for detecting the size of expanded SAMD12 repeats. The median value was selected as the representative number of repeats for further analysis. The results indicated that the median causative TTTCA repeat number ranged from 10 to 647, the median TTTTA + TTTCA repeat number ranged from 526 to 1226, and the proportion of pathogenic TTTCA repeats in the overall repeat sequence ranged from 1.2 to 62.6%. Patient G: II3 had an extremely short TTTCA insertion (10 TTTCA repeat units), which explains the indistinguishable signal presented in RP-PCR (Fig. 2C). She presented with CMT onset at age 33 and no seizures. The range of repeat numbers of both TTTTA and TTTCA repeats in different individuals was remarkably variable. The overall distribution of the repeat numbers is displayed in a box plot (Fig. 3A). The repeat element of the expanded allele was visualized to analyze its structure. The [(TTTTA)exp(TTTCA)exp] configuration was the only type of repeat composition in our case series (Fig. 3B), while other reported configurations, including [(TTTTA)exp(TTTCA)exp(TTTTA)exp] and [(TTTTA)exp(TTTCA)exp(TTTTA)exp(TTTCA)exp], were not observed in our case series. We explored the link between the range of instability and visit age, repeat instability and the average repeat number. As a result, repeat instability was related to its own average number (Pearson's, *N* = 36, *R* = 0.61, *P* = 7e−05, Fig. 4A), not the visit age (Pearson's, *N* = 36, *R* = −0.13, *P* = 0.43, Supplementary Fig. 3). The fluctuation amplitude of the expanded repeat increased with an increase in the repeat number of repeats.

**Table 2:** Genetic data of patients

Pedigree: ID	Gender	Onset age^a^	Expanded TTTTA repeat number			Expanded TTTCA repeat number			Total repeats^b^
			Median	Mean	Mode	Median	Mean	Mode	
A: II2	F	60	356	342	367	170	167	171	526
A: II5	F	59	362	346	374	195	190	201	557
A: II6	M	40	347	333	359	193	190	194	540
A: II8	M	46	346	333	358	189	188	190	535
A: III1	F	50	360	347	372	185	183	187	545
A: III2	F	40	354	341	365	183	181	185	537
A: III4	F	30	359	343	374	220	214	225	579
A: III6	F	41	342	328	354	199	197	202	541
B: II1	M	30	626	600	652	367	348	376	993
B: II2	M	20	631	576	673	390	356	448	1021
B: II4	M	30	631	582	666	411	384	467	1042
B: II7	F	8	624	581	656	403	375	454	1027
B: III1	M	20	671	636	707	525	481	570	1196
B: III2	M	18	671	621	249	552	523	603	1223
C: IV4	F	50	667	643	689	286	291	269	953
C: IV5	M	27	647	619	664	303	298	327	950
C: IV7	M	30	655	627	672	357	345	348	1012
C: IV11	M	27	526	493	551	396	373	408	922
C: IV14	F	37	626	599	650	305	302	304	931
C: IV17	M	35	274	303	285	459	420	473	733
C: IV19	F	40	643	600	660	347	328	354	990
C: V9	F	30	680	626	695	352	333	374	1032
C: V11	M	21	678	622	700	374	344	410	1052
C: V12	M	28	670	625	698	314	303	315	984
C: V15	M	18	532	500	559	409	387	412	941
C: V19	M	15	667	633	699	407	391	410	1074
D: II3	F	66	706	676	717	315	318	313	1021
D: II7	M	50	634	689	609	325	319	314	959
D: III1	F	47	772	732	783	433	407	17	1205
E: II1	M	46	772	706	787	217	211	221	989
E: III1	F	20	869	786	888	252	242	278	1121
F: II2	F	27	580	547	595	588	521	61	1168
F: III5	M	27	573	533	594	517	517	821	1090
F: III6	F	19	542	515	553	573	541	603	1115
F: III7	M	28	579	546	607	647	576	705	1226
G: II3	F	33	819	723	857	10	10	9	829

^a^Age at onset of the first symptom.

^b^Sum of the median TTTTA and TTTCA.

### Genotype-phenotypic correlations

To explore the relationship between repeat expansion and clinical features, we conducted correlation analysis between repeat size and age at disease onset in our case series. We first counted the onset age of CMT and GTCS separately and determined the younger age as the onset age of the disease in our case series. Pearson’s correlation coefficient analysis revealed a significant inverse correlation between the median length of TTTCA and AO (Pearson's, *N* = 36, *R* = −0.52, *P* = 0.0011; Fig. 4Bi), and between the median length TTTTA/TTTCA and AO (Pearson's, *N* = 36, *R* = −0.51, *P* = 0.0014, Fig. 4Bii). An earlier onset age was linked with a larger number of TTTCA repeats. No correlation was found between the length of TTTTA and age at onset (Pearson's, *N* = 36, *R* = −0.3, *P* = 0.078, Fig. 4Biii).

To explore the anticipation of our patient group, we studied the clinical and genetic data of 14 parent-child pairs (7 maternal pairs and 7 paternal pairs) (Supplementary Tables 3 and 4). Clinical anticipation was observed in 13 parent-child pairs involving both symptoms of tremor and seizure, with 11 pairs (6 maternal pairs and 5 paternal pairs) in tremor and 4 pairs (3 maternal pairs and 1 paternal pair) in seizure in our case series. Although anticipation of a higher degree of maternal transmission was observed, the analysis was statistically insignificant in both CMT (*N* = 14, Fisher's exact test, *P* = 1) and GTCS (*N* = 14, Fisher's exact test, *P* = 0.5594). The average anticipation of tremor and seizure was 20 (9–29) and 37 (27–47) years, respectively. We compared the average years of anticipation for symptoms of tremor or seizure between the two transmissions. The results revealed no significant difference in the occurrence of anticipation in different transmissions (*N* = 13, *t*-test, *P* = 0.9349). To evaluate the instability of TTTCA/TTTTA repeat sizes by generation, we studied the change in repeat length in parent-offspring pairs. A change in repeat size was found in all pairs with 13 increases and 1 decrease in total repeat (12 increases and 2 decreases in TTTCA repeat), resulting in an average change of 0.45 kbp (range: −0.39 to 1.7 kbp). No significant difference was identified in the intergenerational dynamic expansion of repeats between maternal and paternal transmissions in either TTTCA repeats (*N* = 14, *t*-test, *P* = 0.8331) or total repeats (*N* = 14, *t*-test, *P* = 0.1653). To clarify the genetic and clinical anticipation, we analysed the relationship between parent-offspring differences in repeat sizes within *SAMD12* and their changes in AO. The results did not indicate this link for either TTTCA repeats (Pearson's, *N* = 13, *r* = 0.43, *P* = 0.13) or total repeats (Pearson's, *N* = 13, *r* = 0.44, *P* = 0.11).

## Discussion

The insertion of intronic pentanucleotide TTTCA repeats has been reported to cause seven FAME subtypes involving seven genes, with FAME1 (*SAMD12*) being the most common. The correlation between the repeat size and phenotypic variables is an important scientific question to be elucidated. As a newly identified repeat expansion disorder, it is challenging to obtain precise sequence information of the repeat elements because of their complexity. RP-PCR, LR-PCR and Southern blotting are reliable techniques with their own advantages in the initial screening of FAME. Targeted LRS or whole-genome LRS is an ideal method for completely clarifying the repeat structure. In a previous study, Cas9-mediated enrichment of LRS was used to elucidate the genomic structure of FAME.^8^ In the present study, we provide an alternative route to analyze knowledge of the size, configuration, and composition of repeats. Enrichment of target region was a critical step for targeted LRS. Compared to CRISPER-cas9 technology, long PCR is uncomplicated, inexpensive and easy to realize a high-depth sequence, while PCR induced bias is an underlying impact. We used combined RP-PCR and long PCR-based high-depth accurate circular consensus sequencing to explore the clinical and genetic characteristics of a Chinese FAME case series.

The included 36 patients in our case series were clinically diagnosed with FAME and genetically identified as FAME1. Typical symptoms of CMT and GTCS with a family history are easy to recognize for FAME. In our case series, clinical features varied among patients, both within and between families. These characteristics are consistent with previous studies.^14^ There were only two subtypes including FAME1 and FAME6 reported in Chinese mainland now.^9,11,12,15-17^ In addition to FAME1, other subtypes are very rare and present a distribution of ethnic backgrounds. The causative repeat elements in the 36 patients were precisely sequenced using the PacBio HiFi platform. The ultra-high sequence depth provided sufficient read coverage for repeat expansion screening and mosaic analysis, thus exploring the nature of the repeat structure. First, the remarkably unstable repeats, including polymorphic TTTTA and pathogenic TTTCA repeats, presented an approximately normal distribution. This repeat instability may be an actual reflect of somatic misaims as this phenomenon had been observed in multiple studies.^6,8,10^ The ultra-high sequencing depth may have amplified the detection of this variability. Based on our results from PCR-free nanopore whole-genome sequencing (WGS), we propose that somatic mosaicism is the most plausible explanation for this observation. However, we could not completely rule out experimental artefacts due to the introduction of PCR technology. Further studies are needed to strengthen the validity and reliability of our genetic approach. Second, the average TTTCA repeat number of our case series ranged from 10 to 647, accounting for 1.2–62.6% of the overall repeat sequence. Ten was the smallest TTTCA repeat number reported for the Chinese FAME. This individual example illustrated that the TTTCA insertion itself was the pathogenic key again,^8,18^ regardless of its size. Third, we found that the fluctuation amplitude of the expanded repeat increased with increasing average number of repeats. This result indicates that a larger number of repeats is more prone to instability during cell proliferation. Finally, the analysis of repeat composition indicated that [(TTTTA)exp(TTTCA)exp] was the only configuration type in our case series. This result indicates that other configurations were rarer in the Chinese population although they were occasionally reported in other published documents.^19^

The correlation between the size of *SAMD12* repeats and clinical variables remains inconclusive.^1,8-10^ Our results revealed a significant inverse correlation between the median length of TTTCA, and between the median length TTTTA/TTTCA and age at onset of either CMT or GTCS. The length of the expanded repeat is thought to correlate with disease severity and the AO in most dynamic mutation disorders. As for FAME, no unified conclusion has been reached in several existing studies on this genotype-phenotypic correlation. Possible reasons include the limited number of samples and the complex repeat structure. The relationship between repeat expansion and clinical features is complex, and further studies with larger sample patient groups are needed. In addition, we observed clinical anticipation in our families. However, we did not find a significant difference in the occurrence of anticipation or the difference in intergenerational dynamic expansion of repeats between maternal and paternal transmissions. We also did not demonstrate a link between parent-offspring differences in repeat sizes and their changes in AO. Our study could not form this conclusion, possibly due to the small sample size of parent-offspring pairs.

## Conclusion

We provided a detailed phenotypic and genotypic analysis of the FAME1 case series from mainland China based on the elucidation of the exact genetic information of the TTTTA/TTTCA repeat expansion using high-depth targeted LRS technologies. We found ten as the smallest causative TTTCA repeats in the Chinese population and an inverse correlation between the onset age and the number of TTTCA repeats and the total number of TTTTA/TTTCA repeats. These findings will provide more evidence regarding the clinical and genetic features of FAME, thus helping to gain a deeper understanding of FAME.

## Supplementary Material

fcaf214_Supplementary_Data

## Data Availability

The sequencing data for the expanded *SAMD12* repeats are available upon reasonable request.
