# Supplementary material for: Phenotypic and genotypic characterization of familial adult myoclonus epilepsy in a Chinese case series
Source: Brain Commun. 2025 Jun 4;7(3):fcaf214. doi: 10.1093/braincomms/fcaf214 (PMC12152537; doi:10.1093/braincomms/fcaf214)
Supplement: fcaf214_Supplementary_Data [file fcaf214_supplementary_data.docx]

1. Supplementary Figure 1


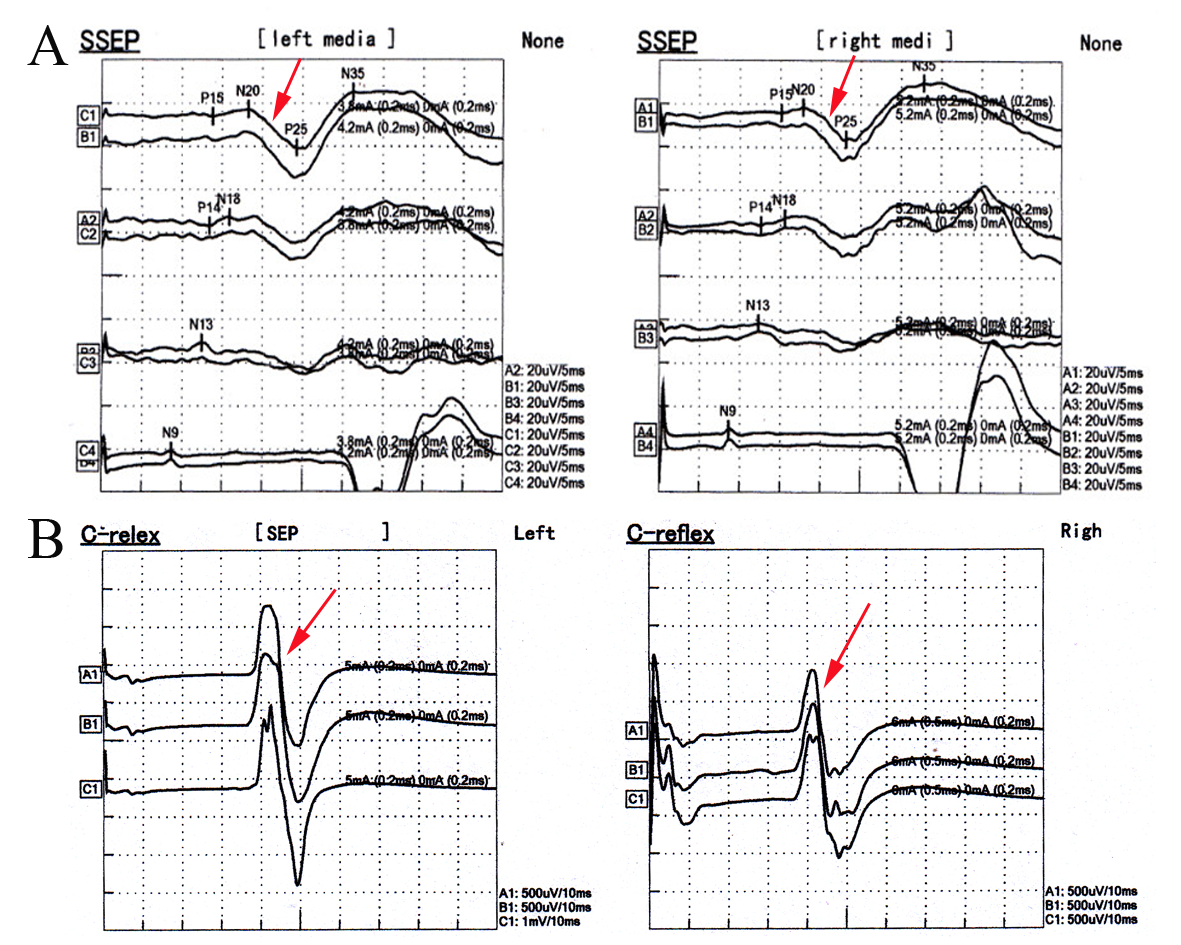


*Representative electrophysiological test results: SEPs indicated enhanced cortical components (N20-P25amplitude >10 µV and enhanced C-flex) by stimulating the median nerve in the bilateral wrist (red arrow indicated). SEP: somatosensory evoked potentials.*

2.Supplementary Figure 2


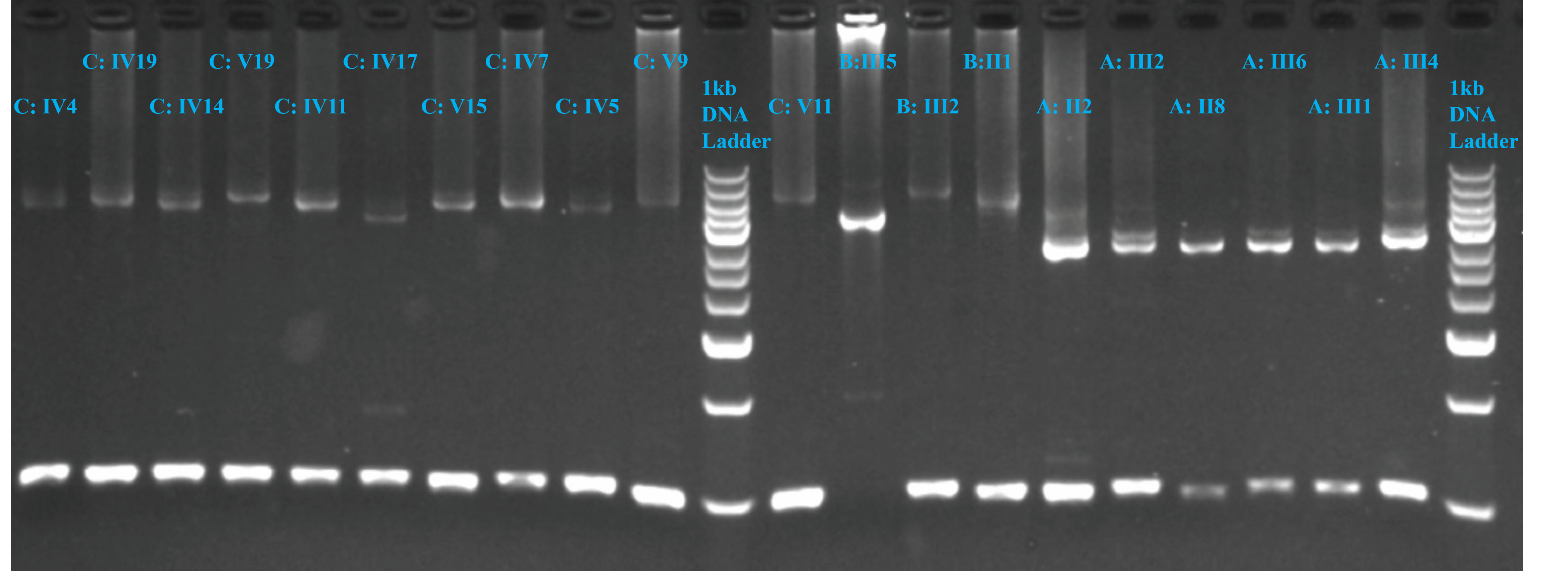


*Representative results of gel electrophoresis of LR-PCR product. Abnormally large PCR products with sizes ranging from 3 to 10 kb. DNA marker: 1 kb DNA Ladder. The lanes were labeled as pedigree: ID. Lane B: III5 which was a presymptomatic carrier, was excluded in further analysis.*

3. Supplementary Figure 3


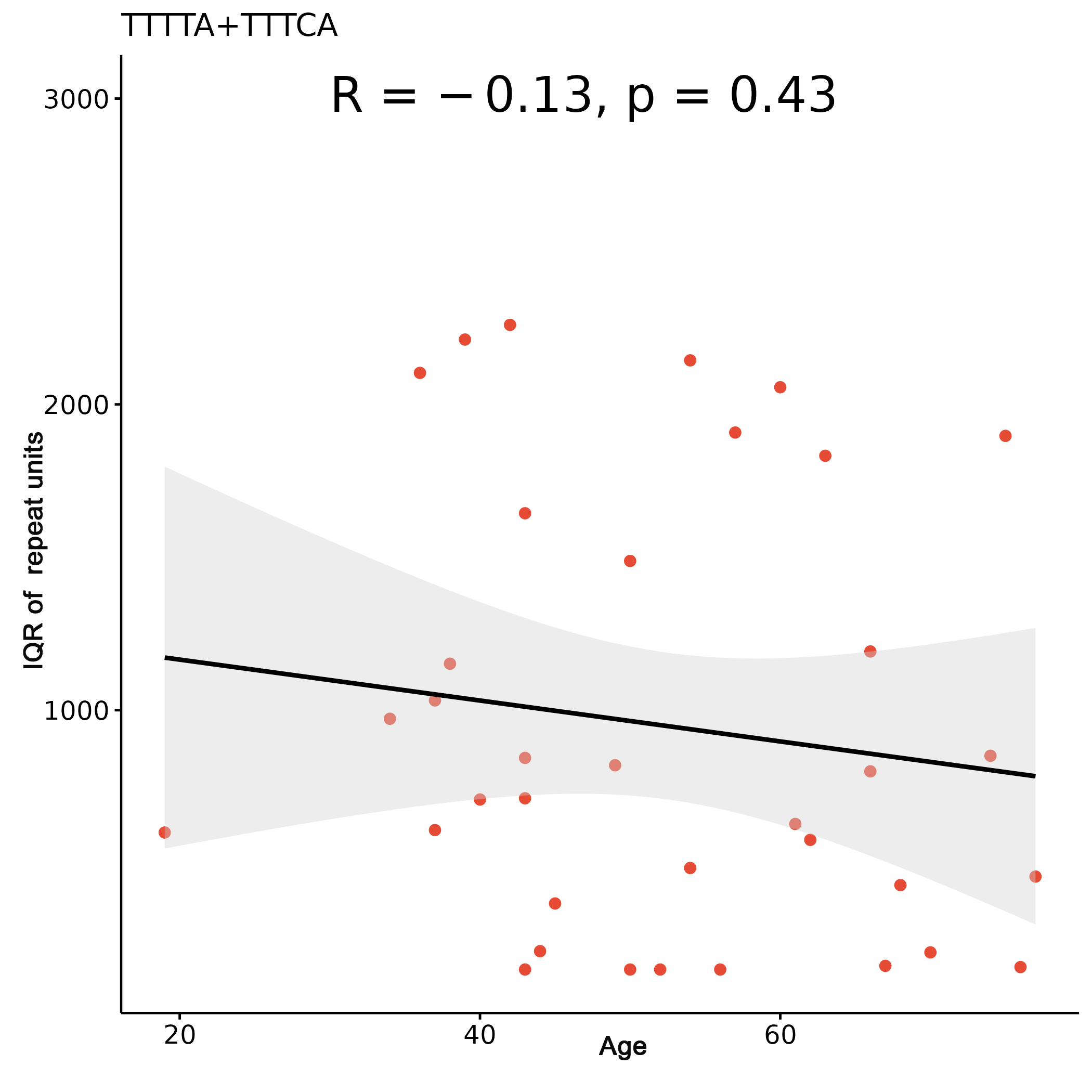


*Scatter plot and linear regression of correlation analysis. Pearson’s correlation analysis indicated that repeat instability was not related to visit age (Pearson’s, N=36, R=-0.13, p=0.43). Each data point represents the repeat number difference of a sample, calculated as the interquartile range (IQR) of the total expanded TTTTA/TTTCA repeats.*

4. Supplementary table 1

Repeat size distribution in reads from long-read sequencing data.

| **Pedigree: ID** | **Expanded repeat number in sequence reads** | | | | | | | | | |
| --- | --- | --- | --- | --- | --- | --- | --- | --- | --- | --- |
|  | **Read 1** | **Read 2** | **Read 3** | **Read 4** | **Read 5** | **Read 6** | **Read 7** | **Read 8** | **Read 9** | **Read 10** |
| a: II2 | 515 | 520 | 522 | 529 | 539 |  |  |  |  |  |
| a: II5 | 413 | 542 | 555 | 560 | 574 | 574 | 582 |  |  |  |
| a: II6 | 493 | 514 | 518 | 527 | 534 | 535 | 536 | 538 | 539 | 548 |
| a: II8 | 505 | 515 | 537 | 538 | 543 |  |  |  |  |  |

5. Supplementary table 2

Representative repeat number of expanded SAMD12 repeats from whole-genome long-read sequencing data. ^a^ Age at onset of the first symptom, ^b^ Sum of the median TTTTA and TTTCA. ^c^ the number difference of TTTTA+TTTCA repeat between the two methods.

| **Pedigree: ID** | **Expanded TTTTA repeat number** | | | **Expanded TTTCA repeat number** | | | **Total repeats^b^** | **delta-expRepeat^c^** |
| --- | --- | --- | --- | --- | --- | --- | --- | --- |
|  | **Median** | **Mean** | **Mode** | **Median** | **Mean** | **Mode** |  |  |
| a: II2 | 354 | 355 | 353 | 168 | 170 | 171 | 522 | 4 (0.8%) |
| a: II5 | 363 | 347 | 354 | 197 | 196 | 194 | 560 | 3(0.5%) |
| a: II6 | 342 | 331 | 329 | 192 | 197 | 201 | 534 | 6(1.1%) |
| a: II8 | 346 | 335 | 340 | 191 | 193 | 188 | 537 | 2(0.4%) |

6. Supplementary table 3

Anticipation and repeat instability of parent-offspring pairs. /: symptoms not reported.

| **Pedigree** | **Parent-offspring** | **Transmission type** | **Anticipation (years)** | | **Delta-median repeat expansion** | | |
| --- | --- | --- | --- | --- | --- | --- | --- |
|  |  |  | **CMT** | **GTCS** | **TTTTA** | **TTTCA** | **Total** |
| **a** | II2-III1 | maternal | 10 | / | 4 | 15 | 19 |
|  | II2-III2 | maternal | 20 | / | -2 | 13 | 11 |
|  | II5-III4 | maternal | 29 | 42 | -3 | 25 | 22 |
|  | II6-III6 | paternal | -1 | / | -5 | 6 | 1 |
| **b** | II2-III1 | paternal | 20 | 35 | 40 | 135 | 175 |
|  | II2-III2 | paternal | 22 | 37 | 40 | 162 | 202 |
| **c** | IV4-V9 | maternal | 20 | / | 13 | 66 | 79 |
|  | IV4-V11 | maternal | 29 | 37 | 11 | 88 | 99 |
|  | IV5-V12 | paternal | -1 | 40 | 33 | 11 | 44 |
|  | IV11-V15 | paternal | 9 | / | 6 | 13 | 19 |
|  | IV17-V19 | paternal | 20 | / | 393 | -52 | 341 |
| **d** | II3-III1 | maternal | 19 | 47 | 66 | 118 | 184 |
| **e** | II1-III1 | paternal | 26 | / | 97 | 35 | 132 |
| **f** | II2-III5 | maternal | / | 27 | -7 | -71 | -78 |

7. Supplementary table 4

Difference in anticipation of parent-offspring pairs. ^a^ symptom reported in both the affected parent and offspring was counted, exp: expansion, n: number, SD: standard deviation.

|  | Total | | Maternal | | Paternal | |
| --- | --- | --- | --- | --- | --- | --- |
|  | CMT | GTCS | CMT | GTCS | CMT | GTCS |
| Transmissions, n | 13^a^ | 6^a^ | 6 | 3 | 6 | 3 |
| Anticipation, n (%) | 11 (84.6%) | 4 (66.7%) | 6 (100%) | 3 (100%) | 5 (83.3%) | 1 (33.3%) |
| Year of anticipation (Mean ± SD (range)) | 20.4±5.8(9-29) | 37.9±5.8(27-47) | 20.2±6.7(10-29) | | 21.8±7.1 (9-28) | |
| Total repeat expansion, n (%) | 13 (92.9%) | | 6 (85.7%) | | 7(100%) | |
| TTTCA repeat expansion, n (%) | 12 (85.7%) | | 6 (85.7%) | | 6(85.7%) | |
| Delta-expTTTTA/TTTCA expansion (Mean ± SD (range)) | 89.3±66.8 (-78-341) | | 48.0±82.5(-78-184) | | 130.6±112.2(1-341) | |
| Delta-expTTTCA (Mean ± SD (range)) | 40.29±108.41 (-71-162) | | 36.3±40.0(-71-118) | | 44.3±61.7(-52-162) | |
